# Fluoroscopy Exposure During Distal Radius Fracture Fixation: An Audit of Current Practice

**DOI:** 10.7759/cureus.99719

**Published:** 2025-12-20

**Authors:** Tarik Al-Dahan, Siddharth Virani, Aliye Nazli Asardag, Nabil Seoudi, Idahosa Izedonmwen, Nima Sherpa, Muhammad Almas Murad, Mutmainah Ekungba-Adewole, Salih Seidahmed, Yogan Gurung

**Affiliations:** 1 Trauma and Orthopaedics, East Kent Hospitals University NHS Foundation Trust, Ashford, GBR; 2 Trauma and Orthopaedics, William Harvey Hospital, Ashford, GBR; 3 Trauma and Orthopaedics, Maidstone and Tunbridge Wells NHS Trust, Royal Tunbridge Wells, GBR

**Keywords:** distal radius fractures, dose-area product, fluoroscopy safety, fluoroscopy time, intraoperative fluoroscopy

## Abstract

Background

Distal radius fractures (DRFs) are among the most common fractures in adults. Volar plate fixation is a widely used treatment. Intraoperative fluoroscopy is essential for confirming reduction and implant positioning, but it exposes patients and staff to ionising radiation. This Study was conducted to assess local practice, quantify radiation exposure, and help to establish national benchmarks for fluoroscopy usage and radiation exposure to improve patients' and staff safety.

Methods

A retrospective review of 106 consecutive patients undergoing volar plate fixation for DRFs was performed in William Harvey Hospital, Ashford, Kent, UK. Demographics, fracture type, surgical details, and intraoperative fluoroscopy parameters were collected, including screening time, number of exposures, dose area product (DAP), and air kerma (AK). Data were analysed by fracture type and surgeon grade.

Results

The cohort included 31 males and 75 females, with a mean age of 59 years. Most fractures were intra-articular (n = 93). All patients underwent volar plate fixation. The mean operative time was 73 minutes. Fluoroscopy exposure during distal radius fixation demonstrated a mean screening time of 24 seconds (range 0.1-111), with a mean of 43 exposures (range 5-209), a mean dose area product (DAP) of 4.75 µGy·m² (range 0.31-29.26), and a mean AK of 0.26 (range 0.01-9). Further subgroup analysis was performed to quantify exposure by surgeons' grade and fracture classification.

Conclusion

Intraoperative fluoroscopy during volar plate fixation of DRFs in our institution resulted in a mean DAP of 4.75 µGy·m² and a mean screening time of 24 seconds. No significant differences were observed by operator grade or fracture complexity. Future efforts should focus on standardising its collection and defining orthopaedic Diagnostic reference levels. Regular audit of fluoroscopy usage against established standards would help in improving uniformity across hospitals and orthopaedic teams.

## Introduction

Distal radius fractures (DRFs) are common injuries; they account for 18% of all fractures in the elderly population [1]. Operative fixation with volar plating is a common option to treat these fractures in the adult population in order to restore the function of the wrist and to prevent long-term complications [2]. Fluoroscopy plays an essential role in confirming fracture reduction and implant placement. However, intraoperative fluoroscopy use places patients and staff at risk of ionising radiation [3,4]. Although there have been attempts to standardise and acknowledge the ionising radiation doses in various fluoroscopy-guided procedures [5], knowledge gaps among medical and theatre staff still exist, with a lack of long follow-up data. 

Many parameters have been used to measure the radiation exposure, and current fluoroscopy machines have complex adjustments to optimise the fluoroscopy images. It became well known that the fluoroscopy time is not the sole factor in determining the radiation exposure and, in fact, is the least useful parameter to use to estimate the risk [6]. Kerma is the amount of radiation per unit mass, and the air kerma (AK) is the amount of radiation released from the X-ray beam at a specific point in the air. It measures the intensity of the X-ray beam. Dose area product (DAP) is the product of AK multiplied by the area of the beam; it measures the total radiation dose the patient received [6]. Most fluoroscopy machines do not measure skin exposure directly. However, there is a strong correlation between DAP and the maximum radiation skin exposure [7].

Radiation safety in the UK is regulated by the Ionising Radiation (Medical Exposure) Regulations (IR(ME)R)[8]. It identifies the justification for exposure, optimisation, and responsibilities of the healthcare professionals to minimise the risks associated with the ionising radiation. National diagnostic reference levels have been identified for the common procedures by the UK Health Security Agency (UKHSA) Medical Dosimetry Group (MDG) [9]. However, there is a lack of standardisation of the orthopaedic procedures.

In vascular surgery, DAP values are monitored, which are then benchmarked against the diagnostic reference levels (DRLs). This, in return, allows national comparison and a dose-alert system. Orthopaedics lacks this unified framework for reporting and auditing radiation data. This can also be viewed from the limited number of studies being published on this matter, especially from the UK. Emphasis on this has already been put on this by Umanes et al. [10], who showed that less than one-third of UK orthopaedic units perform regular dosimetry review, and formal radiation safety education remains inconsistent. Understanding the confounders of DAP and standardising DAP reporting within the operative notes or in the picture archiving and communication system (PACS) could enable more robust benchmarking. McAleese et al. [11] created a standardised communication tool that has shown in their results to reduce patient and staff radiation exposure.

Advances in the current image intensifiers, or at least the ones used in our hospital, allow the operator to adjust many fluoroscopy variables such as the frame rate, radiation intensity, and image magnification with the ability to record the exposure parameters and save them on the PACS system for each patient, allowing accurate auditing and analysis. This paper aimed at identifying the amount of radiation exposure for a commonly performed orthopaedic procedure of distal radius fracture fixation by volar plating, in which fluoroscopy usage is inevitable, which could be used as a foundation for new guidelines and standards for patients' and staff safety.

## Materials and methods

A single-centre audit was registered with the institutional audit and governance review board of East Kent University Hospitals NHS Foundation Trust (RN1746809). Data collection was performed retrospectively, from January 2023 to September 2025, at William Harvey Hospital, Ashford, Kent, UK. We identified adult DRFs treated by open reduction and internal fixation with volar plating using our theatre list software, Theatreman (Trisoft Ltd, UK), theatre management system. Data were extracted from the patients' records into an Excel sheet (Microsoft Excel, Microsoft Corp., USA) for further analysis. The fluoroscopy report for each patient includes information about the fluoroscopy time, DAP, number of exposures, and modes of fluoroscopy.

The primary outcomes were to assess the number of exposures, fluoroscopy time, and DAP. The secondary outcomes were the relation between the number of exposures and DAP, the relation between the fracture type (intra-articular versus extra-articular) and radiation exposure, and the relation between the primary surgeon’s grade and exposure. Descriptive statistics and histogram inspection in Microsoft Excel (Microsoft Excel for Microsoft 365 MSO (version 2509, build 16.0.19231.20138, 64-bit ) showed that the screening time, number of exposures, and dose area product were skewed and not normally distributed. Therefore, a non-parametric test (the Mann-Whitney U test) was used for inter-group comparison. The test was performed in Numiqo [12] with the calculation of the effect size (r).

This was a retrospective single-centre audit for service evaluation; therefore, no a priori sample size calculation was undertaken. Instead, the sample size was determined by including all eligible distal radius cases within the specified time frame to avoid bias. We identified 145 cases; however, 39 cases were excluded as no fluoroscopy data were saved in the patients' records, which renders them of no value for analysis. Inclusion criteria were adults with DRFs treated by open reduction and internal fixation using a volar plate. Exclusion criteria were paediatric fractures, multiple-injured patients and fractures fixed with other fixation methods rather than a volar plate.

## Results

The study included 106 cases of DRFs, of whom 31 were males and 75 were females. The mean age for the included cohort is 59 years. Fluoroscopy data were pooled and analysed. Intra-articular fractures were the majority of the cases (n = 93), with the remainder being extra-articular (n = 13). All fractures were fixed with a volar locking distal radius plate. The mean operative time was 73 minutes (range 17-183). Most of the operations were performed by registrars (n = 78), with consultants performing the rest of the cases (n = 28).

**Figure 1:**
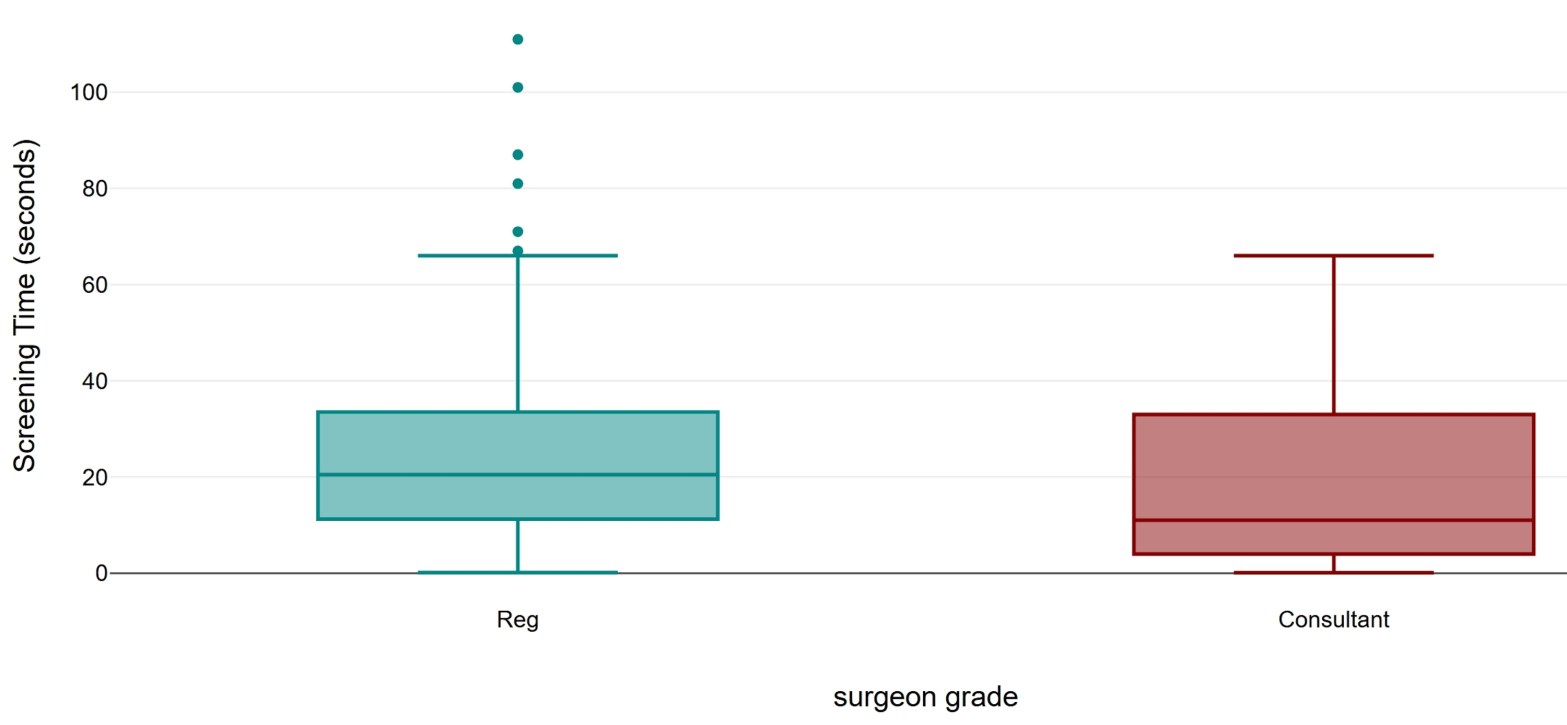
Comparison of screening time by surgeon's grade.

**Figure 2:**
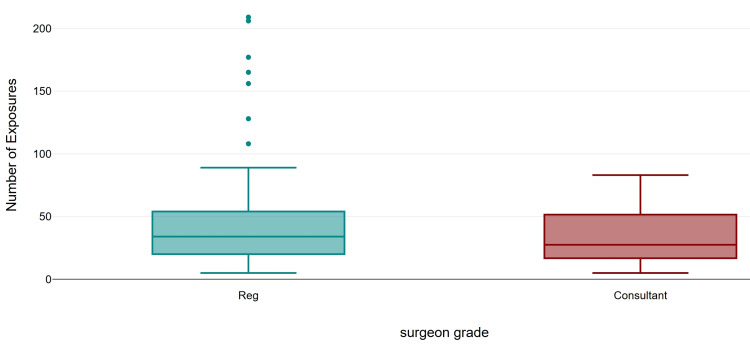
Comparison of number of exposures by surgeon's grade.

**Figure 3:**
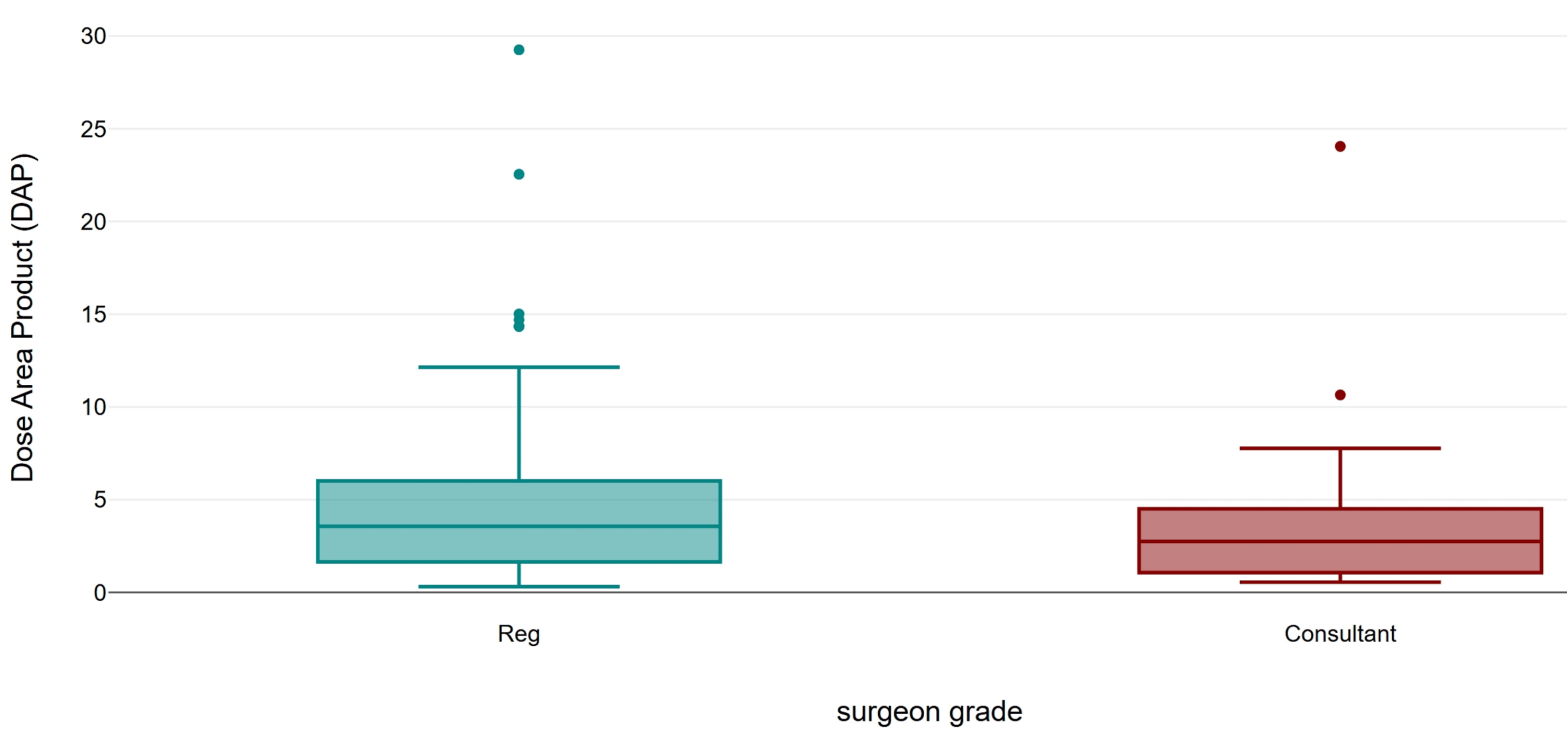
Comparison of dose area product by surgeon's grade.

Regarding fluoroscopy parameters, the mean number of exposures was 43 (range 5-209), the mean screening time was 24 seconds (range 0.1-111), the mean DAP was 4.75 µGy·m² (range 0.31-29.26), and the mean AK was 0.26 (0.01-9). All cases used pulsed fluoroscopy mode. Further subgroup analysis was performed to stratify exposure by surgeon’s grade and fracture classification. The screen time (mean 26.48 s, SD 22.52; median 20.5 s), number of exposures (mean 46.45, SD 43.50; median 34), and mean DAP (mean 5.05 µGy·m², SD 5.06; median 3.57) were higher in the registrar group compared to consultants (mean 19.80 s, SD 19.25, median 11.0 s; mean 33.75, SD 21.18, median 27.5; 3.95 µGy·m², SD 4.65, median 2.75). However, the difference was not statistically significant for all the three parameters (Mann-Whitney U = 864.0, z = −1.63, exact p = 0.104, r = 0.16; U = 922.5, z = −1.13, exact p = 0.262, r = 0.11; and U = 900.0, exact p = 0.171, r = 0.13, respectively) (Figures 1-3). 

**Figure 4:**
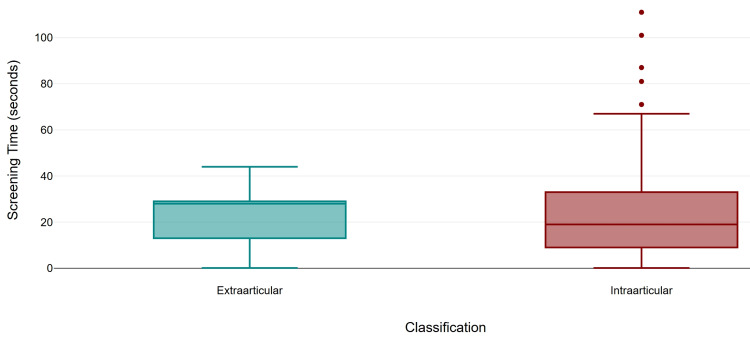
Comparison of screening time by fracture classification.

**Figure 5:**
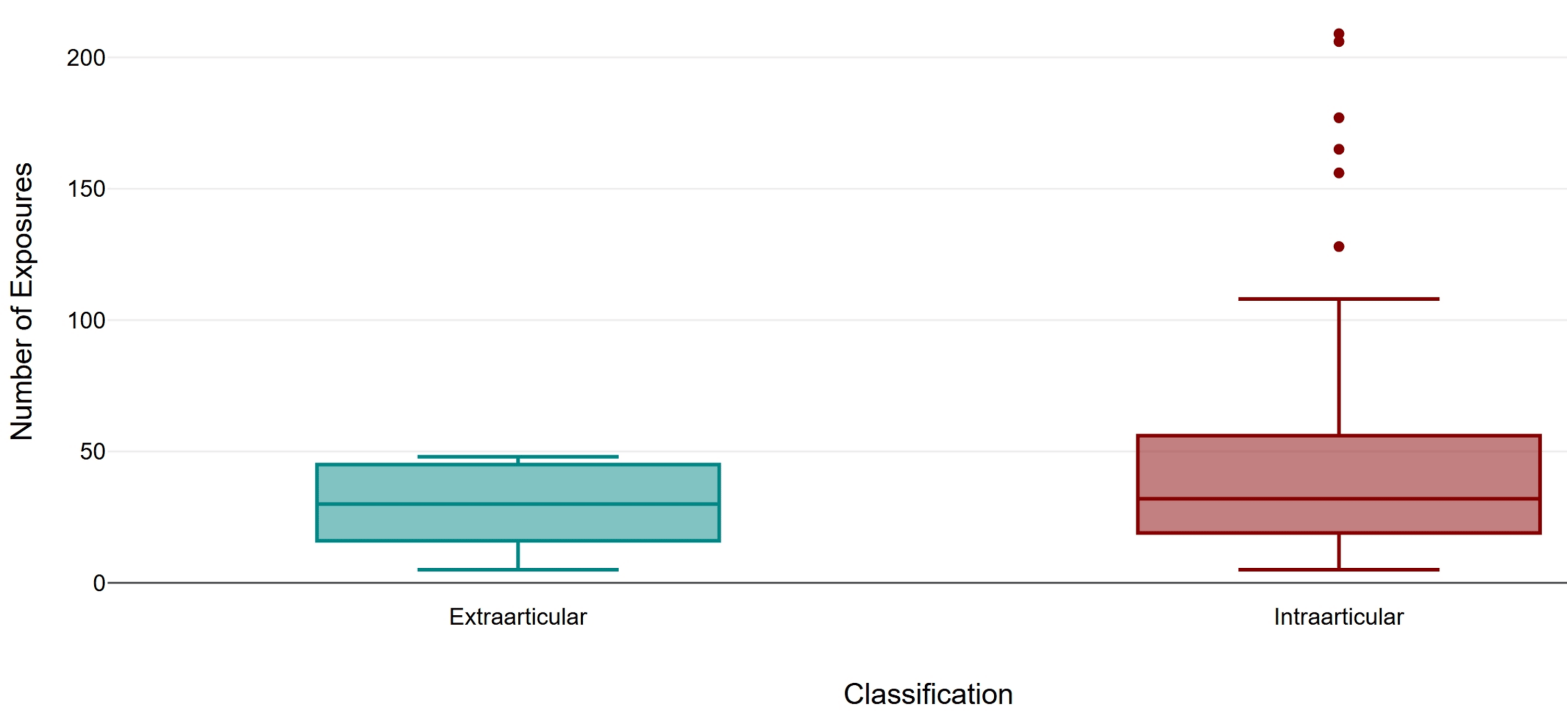
Comparison of number of exposures by fracture classification.

**Figure 6:**
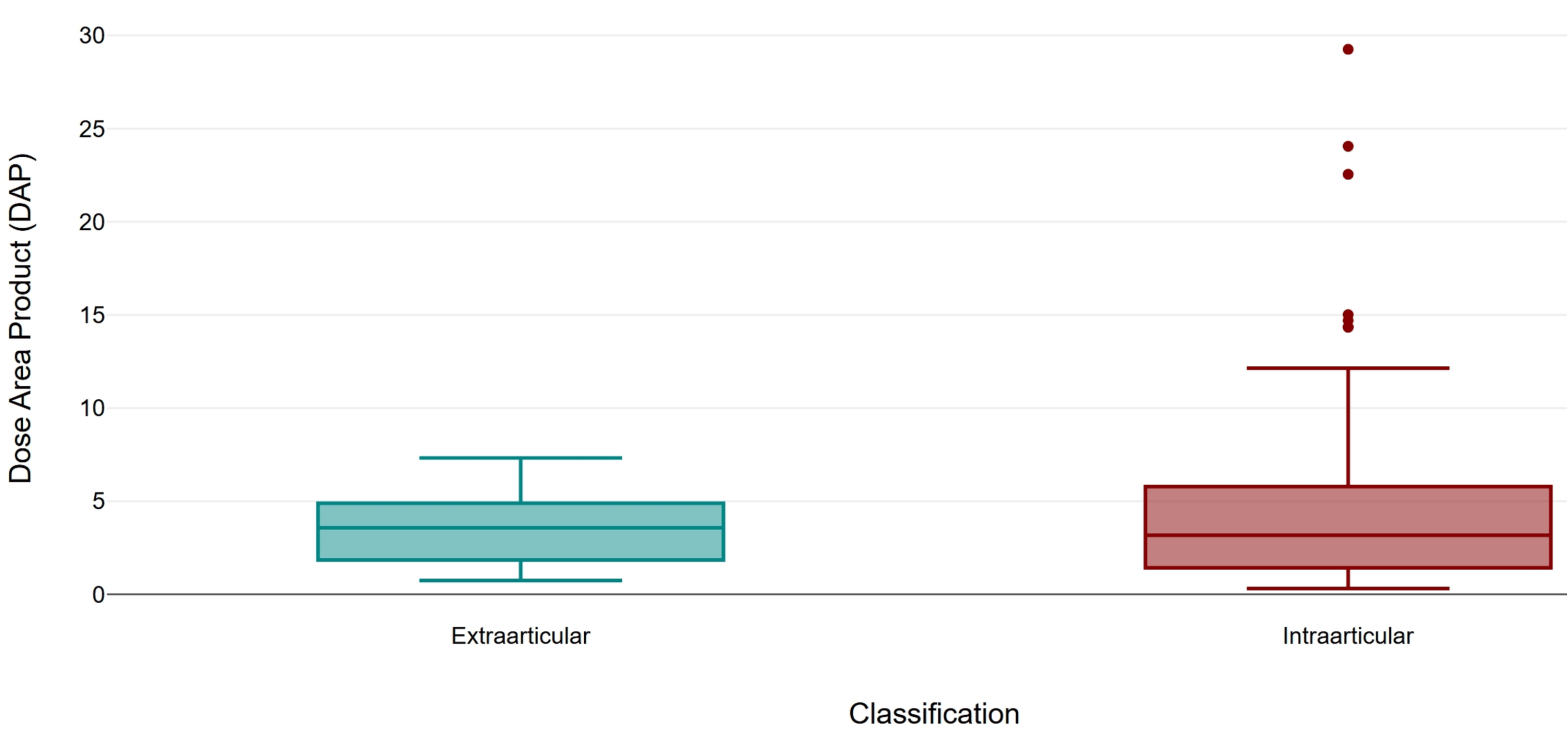
Comparison of dose area product (DAP) by fracture classification.

Screening time was similar between intra-articular fractures (mean 25.07 s, SD 22.81, median 19.0) and extra-articular fractures (mean 22.16 s, SD 12.95, median 28.0). The number of exposures was higher in the Intraarticular fractures (mean 44.93, SD 41.10, median 32) compared to the extraarticular cohort (mean 29.85, SD 15.59, median 30). Intra-articular fractures demonstrated a higher mean DAP (mean 4.92 µGy·m², SD 5.22, median 3.17) compared to extra-articular (mean 3.59 µGy·m², SD 2.11, median 3.57). Similarly, the difference in the parameters between the two groups was not significant (Mann-Whitney U = 590.0, p = 0.893, r = 0.01; U = 501.5, p = 0.351, r = 0.09; and U = 591.5, p = 0.904, r = 0.01, respectively (Figures 4-6).

**Figure 7:**
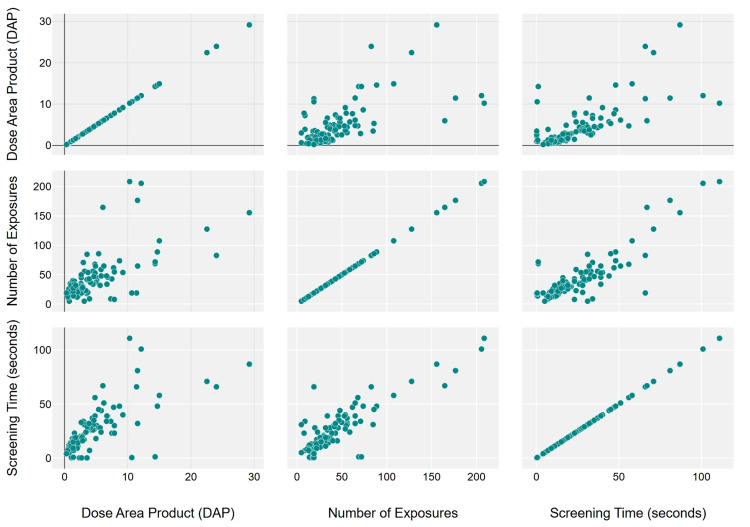
Scatterplot showing the correlation between fluoroscopy parameters.

Spearman correlation analysis was conducted to assess the relationship between fluoroscopy parameters. Our results indicate a strong correlation between DAP and number of exposures (Spearman's correlation coefficient (rₛ) = 0.65, P < 0.001) and between DAP and screening time (rₛ = 0.70, P < 0.001) and similarly between screen time and number of exposures (rₛ = 0.75, P < 0.001) (Figure 7).

## Discussion

In order to best represent radiation exposure, the metric of choice has been widely debated in the field of surgery. In their seminal study, Skripochnik et al. [6] evaluated fluoroscopy time, DAP, and AK in over 145 endovascular procedures. They have concluded that DAP and AK are better surrogates compared to fluoroscopy time, as they can reflect not only time but also the beam intensity and duration. Moreover, both orthopaedic and interventional studies have established DAP as the most robust and universal surrogate for radiation exposure, reflecting not only time but also beam intensity and field size [6,13].

In this study, we have quantified intraoperative fluoroscopy use and radiation exposure during volar plate fixation of DRFs in a single trauma centre through DAP, number of exposures, and fluoroscopy time. As a part of our secondary aims, we looked into how operator grade and the fracture pattern influenced the radiation exposure. Our results largely align with the recent larger-scale study from Liu et al. [14], who have reported median DAP values of 9.24 cGy·cm² (equivalent to 9.24 µGy·m²) across 342 DRF fixations. Although Liu et al. [14] did not solely focus on a single operative measure, the majority of their cases were done as volar plating. The mean DAP and fluoroscopy time recorded in our cohort is in the lower end of the published paper by Metaxas et al. [15], where they recorded dose values ranging between 20.1 and 197 mGy·cm^2^ for DAP and 4.50 and 14.5 seconds for fluoroscopy time.

One of the interesting findings that contrasts with the similar study conducted by Liu et al. [14] was the possible effect of the operator grade and DAP. In our study, although we found that there was a higher mean DAP among the registrar-led operations, this was not statistically significant. In their study, Liu et al. [14] found significantly higher DAPs among junior attendings. There are a few points here to consider. Our smaller cohort size could have resulted in a type II error from a limited sample size. On the other hand, the differences accounted for in our studies could be a reflection of differences in surgical training between different countries. Structured supervision, typical in the UK, whereby there is strong consultant guidance, could have accounted for our finding.

Higher mean DAPs have been reported for increased fracture complexity in previous studies [14]. Although intra-articular fracture patterns had slightly higher mean DAPs in our cohort, this was not a statistically significant finding supported also across other orthopaedic procedures. Increasing radiation dose with fracture complexity has been supported in other orthopaedic procedures, such as paediatric supracondylar fractures. Higher-grade injuries were more closely linked with higher DAP compared to lower-grade fractures [16]. Similarly, more complex and centrally located fracture patterns of the pelvis and femur have been shown to result in greater radiation exposure in comparison to simple or distal extremity fracture patterns [17].

The main limitation of our study has been the sample size, which limits statistical power, particularly with regard to the subgroup analysis. Another limitation was that the measurements were focused mainly on patients' exposure. However, there is scattered radiation to the operating surgeon and theatre staff, which we could not measure due to the retrospective data collection, which could be an area of future work. Nonetheless, our data contributes valuable information which could be used in the formation of modern standards for upper limb fixation. A prospective multi-centre study followed by a meta-analysis would be invaluable for the establishment of DRLs for common orthopaedic procedures and enable us as clinicians to determine safe exposure thresholds both for our patients and our staff.

## Conclusions

Intraoperative fluoroscopy during volar plate fixation of DRFs in our institution resulted in a mean DAP of 4.75 µGy·m² and a mean screening time of 24 seconds. No significant differences were observed by operator grade or fracture complexity. DAP remains the most comprehensive and universally comparable measure of radiation burden across both orthopaedic and vascular domains. Future efforts should focus on standardising its collection, defining orthopaedic DRLs. Regular audit of fluoroscopy usage against established standards would help in improving uniformity across hospitals and orthopaedic teams.
